# Conserved Alternative Splicing and Expression Patterns of Arthropod N-Cadherin

**DOI:** 10.1371/journal.pgen.1000441

**Published:** 2009-04-03

**Authors:** Shu-Ning Hsu, Shinichi Yonekura, Chun-Yuan Ting, Hugh M. Robertson, Youichi Iwai, Tadashi Uemura, Chi-Hon Lee, Akira Chiba

**Affiliations:** 1 Neuroscience Program, University of Illinois, Urbana, Illinois, United States of America; 2 Unit of Neuronal Connectivity, Laboratory of Gene Regulation and Development, National Institute of Child Health and Human Development, National Institutes of Health, Bethesda, Maryland, United States of America; 3 Department of Entomology, University of Illinois, Urbana, Illinois, United States of America; 4 Graduate School of Biostudies, Kyoto University, Kyoto University, Kyoto, Japan; 5 RIKEN Brain Science Institute, Saitama, Japan; 6 Department of Biology, University of Miami, Coral Gables, Florida, United States of America; University of California San Francisco, United States of America

## Abstract

Metazoan development requires complex mechanisms to generate cells with diverse function. Alternative splicing of pre-mRNA not only expands proteomic diversity but also provides a means to regulate tissue-specific molecular expression. The *N-Cadherin* gene in *Drosophila* contains three pairs of mutually-exclusive alternatively-spliced exons (MEs). However, no significant differences among the resulting protein isoforms have been successfully demonstrated *in vivo*. Furthermore, while the N-Cadherin gene products exhibit a complex spatiotemporal expression pattern within embryos, its underlying mechanisms and significance remain unknown. Here, we present results that suggest a critical role for alternative splicing in producing a crucial and reproducible complexity in the expression pattern of arthropod N-Cadherin. We demonstrate that the arthropod *N-Cadherin* gene has maintained the three sets of MEs for over 400 million years using *in silico* and *in vivo* approaches. Expression of isoforms derived from these MEs receives precise spatiotemporal control critical during development. Both *Drosophila* and *Tribolium* use ME-13a and ME-13b in “neural” and “mesodermal” splice variants, respectively. As proteins, either ME-13a- or ME-13b-containing isoform can cell-autonomously rescue the embryonic lethality caused by genetic loss of N-Cadherin. Ectopic muscle expression of either isoform beyond the time it normally ceases leads to paralysis and lethality. Together, our results offer an example of well-conserved alternative splicing increasing cellular diversity in metazoans.

## Author Summary

Animal development requires complex mechanisms to generate many different types of cells. Alternative splicing is a process by which a single gene could produce several protein variants under particular circumstances. It is a useful means to generate a diversified set of proteins in different cell types. In this report, we showed that the alternative splicing of the arthropod *N-Cadherin* gene has been maintained for over 400 million years. The switch of expression patterns of two distinct variants is also well conserved in arthropods. As proteins, these two N-Cadherin splice variants have similar ability to rescue the embryonic lethality caused by genetic loss of N-Cadherin. However, when the expression of either isoforms was prolonged in muscles where the endogenous expression ceased beyond certain stages, it leads to larval lethality, suggesting the importance of precise spatiotemporal regulation of N-Cadherin splice-variant expression. This finding is particularly important because it offers an example of well-conserved alternative splicing increasing cellular diversity in animals.

## Introduction

During early metazoan development, cells undergo a complex process in which they are organized into germ layers that further differentiate into various cell types each serving distinct functions. This process requires cellular complexity resulting from molecular diversity in each cell. The post-genomic era has brought the view that the number of protein-coding genes is insufficient to account for the cellular complexity of multicellular organisms. For example, the *Caenorhabditis elegans* genome contains some 19,000 protein-coding genes [1] whereas the human genome contains no more than 25,000 [2], undermining the simplistic notion that the cellular complexity of an organism rises in proportion to the number of protein-coding genes.

Alternative splicing of pre-messenger RNA drastically increases the molecular complexity of the mRNAs expressed in cells [3]. While only 0.05% of protein-coding genes (3 out of 6000) in *Saccharomyces cerevisiae* are alternatively spliced, the majority of protein-coding genes in the human genome are known to undergo alternative splicing [4]–[9], supporting the significance of alternative splicing in generating molecular diversity in metazoan evolution. Alternative splicing is particularly abundant in the brain [9]. Deficiency in producing precise splicing variants has been implicated in several neurological diseases [10],[11]. Estimated numbers of splice-variants from a single gene range from just two in *C. elegans* Cadherin [12] to approximately 70 with mammalian Protocadherin [13],[14] and over 38,000 with *Drosophila* Dscam [15]. Qualitative differences between individual splice-variant isoforms could allow them to distinguish different protein binding partners [4],[16],[17] or nucleotide binding sequences [18]. Splice variants could also be targeted to separate subcellular domains [19],[20] and receive differential degradation controls [21].

Since alternative splicing might allow faster evolution of protein sequences, one might expect that nucleotide sequences of constitutive exons would be more conserved than those of alternative exons. On the contrary, when analyzing human and mouse orthologous transcriptomes, a higher degree of nucleotide sequence conservation is frequently observed in the alternatively spliced exons and/or flanking introns than in the constitutive exons [22]–[27]. Such incidences are due to the conserved presence of *cis*-acting regulatory elements that interact with the core spliceosomal components and tissue-specific splicing factors [11],[28],[29]. This supports the idea that tissue-specific regulation of alternative splicing is through combinatorial expression of splicing factors. Therefore, in addition to generating proteins of diverse functions, regulated expression patterns of alternative splicing could provide an additional layer of control over the quantity of expression [30]–[33].

Previous work in *Drosophila* shows that Neural Cadherin (N-Cadherin) is expressed in both the nervous system and early mesoderm in embryos. N-Cadherin mediates homophilic cell adhesion, associates with beta-Catenin, and causes neurogenesis defects when genetically deleted [34]–[39]. In this study, we examined alternative splicing of the arthropod *N-Cadherin* gene. By combining (i) *in silico* pan-genomic data-mining, (ii) *in vivo* expression assessment in evolutionarily distant organisms, and (iii) isoform-specific genetic manipulations in model organisms, we found that *N-Cadherin* alternative splicing is conserved and the expression patterns of splice- variants are tightly regulated during arthropod embryonic development.

## Results

### Conserved alternative splicing of arthropod N-Cadherin

**Figure 1:**
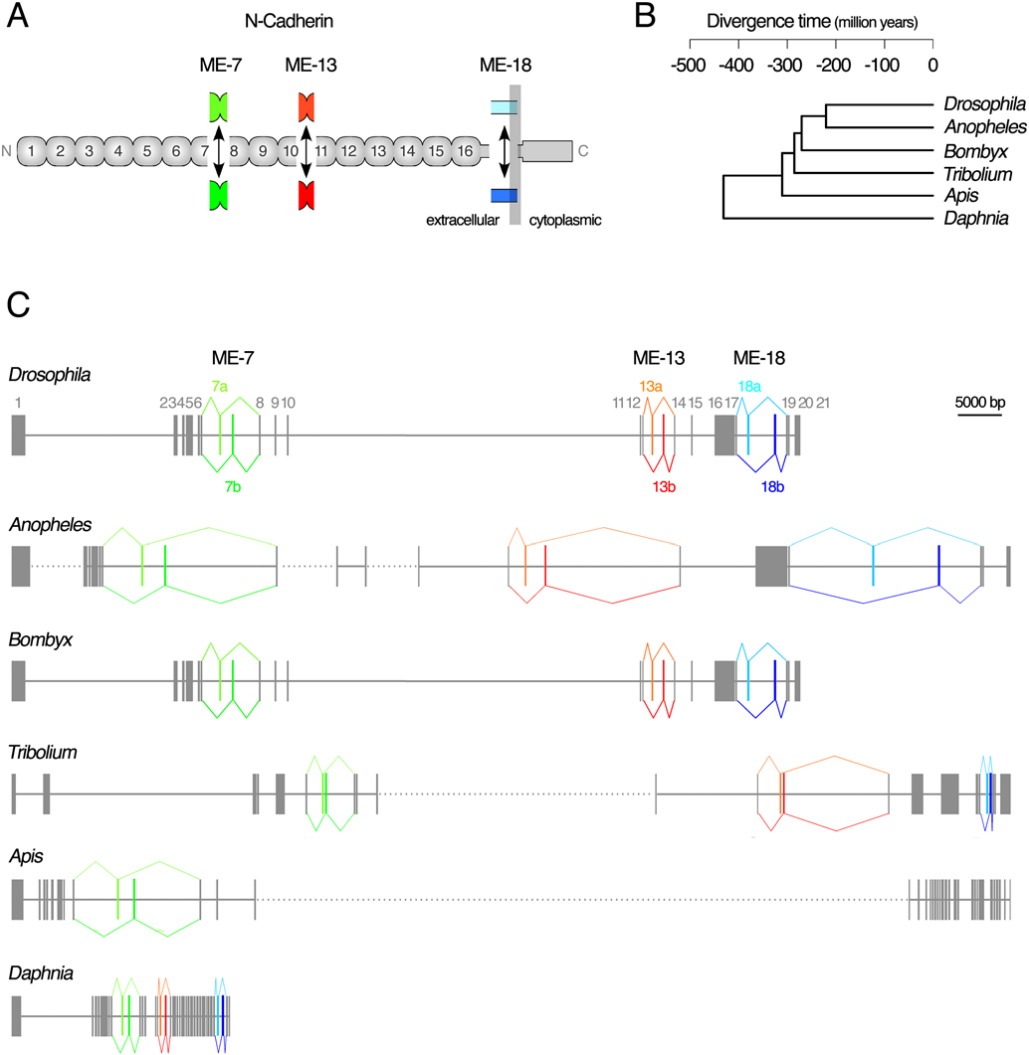
Conserved genomic organization of N-Cadherin in arthropod genomes. (A) Predicted protein structure of arthropod N-Cadherin contains sixteen tandem repeats of extracellular Cadherin domains (*1–16*), a single transmembrane domain, and the ß-Catenin-binding cytoplasmic domain. (B) Phylogenetic tree of the arthropod genomes examined. (C) Genomic organization of N-Cadherin in arthropod genomes exhibits conserved *ME* (mutually-exclusive alternatively-spliced exons)-*7s* (*ME-7a* and *ME-7b*), *ME-13s* (*ME-13a* and *ME-13b*) and *ME-18s* (*ME-18a* and *ME-18b*). Exons are shown as rectangles while introns as horizontal lines, drawn in proportion to their actual length. Common exons are also numbered for *Drosophila*.

Following the release of the *Drosophila melanogaster* genome sequences [40], it became possible to analyze the genomic organization of the *Drosophila N-Cadherin* gene. Based on this analysis, we predicted *Drosophila N-Cadherin* gene to contain three sets of mutually exclusive exons (MEs) within its open reading frame and that it would produce hitherto unknown splice-variants. Combinatorial use of these three sets of *N-Cadherin* MEs, each containing two alternative exons, is predicted to yield up to 8 splice-variant isoforms that form similar structures (Figure 1A and 1C) [41]. (Note: A 12-nucleotide long micro exon-7a' is also predicted in all insect genomes examined in this study. It was always paired with exon 7a, but never 7b, in the mature transcripts).

We examined various genomes outside the *Drosophila* genus to assess the evolution of the genomic organization of the *N-Cadherin* gene. A single *N-Cadherin* ortholog is found in mosquito (*Anopheles gambiae*), silkworm (*Bombyx mori*), red flour beetle (*Tribolium castaneum*), honeybee (*Apis mellifera*), and water flea (*Daphnia pulex*) (Figure 1C) (see Materials and Methods “*N-Cadherin orthologs*”). These insect and crustacean species that contain the *N-Cadherin* gene are, respectively, 230, 270, 290, 300, 430 million years apart from *Drosophila melanogaster* (Figure 1B) [42]. Furthermore, the exact genomic organization of the three sets of MEs is conserved in these arthropod genomes, with a notable exception in *Apis mellifera*. This suggests a recent loss of both ME-13 (exon13b) and ME-18 (exon18b) (Figure 1C) after diverging from other insect species. The amino acid sequences encoded by arthropod *N-Cadherin* genes predict the same overall protein structure (Figure 1A). Considering that the number of common exons and the lengths of interspersing introns vary widely from genome to genome (Figure 1C), the particular genomic stability noted for MEs in *N-Cadherin* implicates their importance for the survival of arthropods.

**Figure 2:**
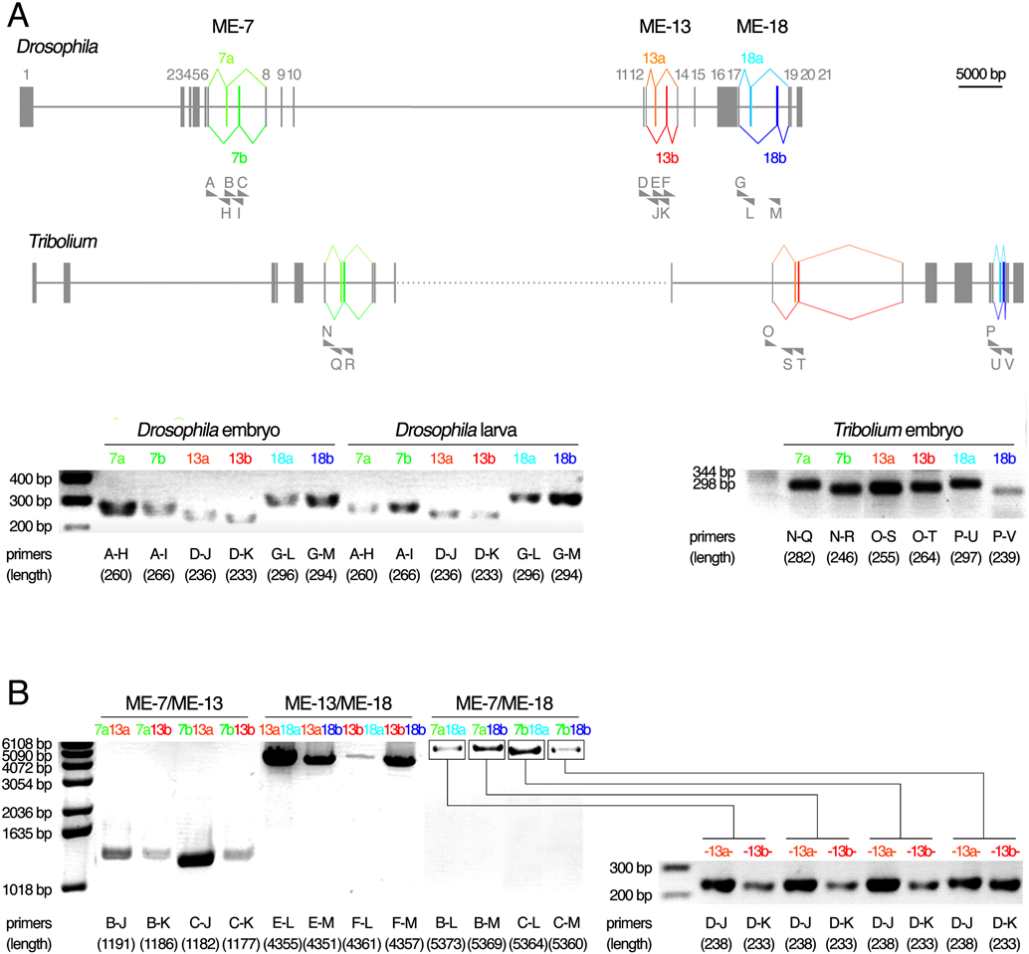
Confirmation of *ME* usage by splice-variants in *Drosophila* and *Tribolium*. (A) RT-PCR detection of all six *MEs* predicted in the genomes of *Drosophila* and *Tribolium*. (B) Nested RT-PCR detection of all eight possible combinations of *ME* usage in *Drosophila* embryos. Orientations and positions of primers (*A-V*) used in Figure 2A and 2B are indicated with semi-arrowheads. The lengths of predicted PCR products are shown in parentheses.

We used RT-PCR to verify the endogenous expression of all proposed MEs of *N-Cadherin* in *Drosophila* and *Tribolium* embryos. The results support at least 290 million years of conserved usage of these MEs (Figure 2A). Using nested RT-PCR, we further confirmed the endogenous expression of each of the eight predicted splice-variant mRNAs in *Drosophila* (Figure 2B). These splice-variants are designated as 7b-13a-18a (the first cDNA described [36]), 7a-13a-18a, 7a-13b-18a, 7b-13b-18a, 7b-13a-18b, 7a-13a-18b, 7a-13b-18b, and 7b-13b-18b. We cloned additional full-length cDNAs from embryos, yielding 7a-13b-18b, 7b-13a-18a, 7a-13b-18a, supporting independently the presence of mRNA splice-variants *in vivo* (data not shown). All endogenous *N-Cadherin* mRNA molecules in embryos utilize MEs that would encode parts of extracellular Cadherin (EC) and transmembrane domains (Figure 1A). With the evolutionarily conserved genomic organization, the arthropod N-Cadherin genes offer an opportunity to evaluate the significance of alternative splicing through *in silico* and *in vivo* analyses.

### Functional redundancy of *Drosophila* N-Cadherin splice-variants

**Figure 3:**
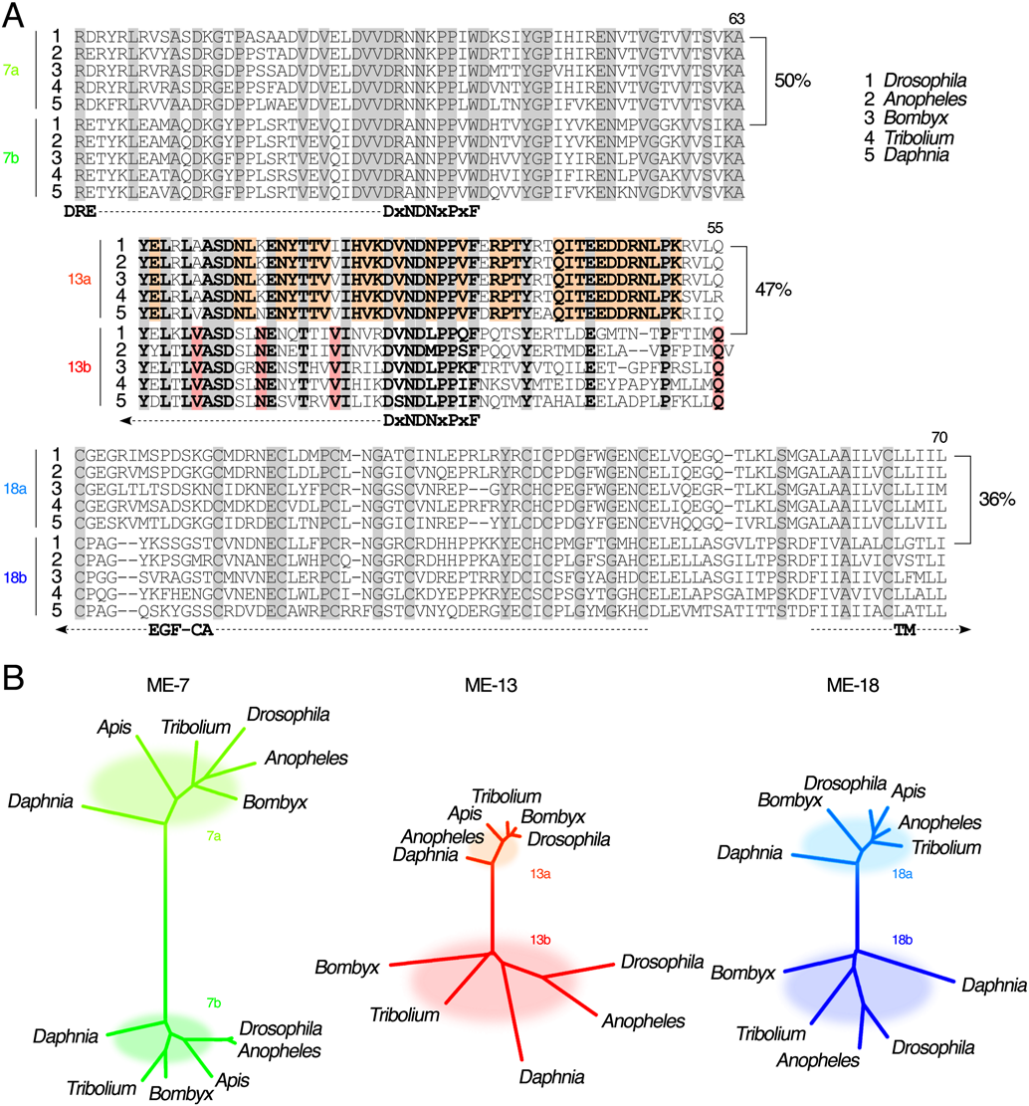
Conservation of amino acid sequences encoded by MEs. (A) Predicted amino acid sequences derived from MEs in five arthropod genomes. Conserved amino acid sequences for ME-13 pairs are shown in bold. Some occur as mutually exclusive for ME-13a (in the orange background) or for ME-13b (in the red background). Amino acid identities (%) between alternative pairs of MEs in *Drosophila* are indicated. Lengths of *ME-7's*, *ME-13's* and *ME-18's* are, respectively, 63, 54–55 and 68–70 amino acids. *DRE* and *DxNDNxPxF* constitute putative Ca^2+^-binding motifs. EGF-like Ca^2+^-binding cystine-rich (*EGF-CA*) and transmembrane domains are indicated. (B) Phylogenetic trees of the MEs based on predicted amino acid sequences. *Apis* lacks MEs for *ME-13s* and *ME-18s*, but has single exons that resemble, respectively, *ME-13a* and *ME-18a*. While each tree is drawn to scale, the branch lengths from different trees are not comparable.

Predicted amino acid sequences encoded by MEs in N-Cadherin of arthropod genomes are highly conserved (Figure 3A). The putative Ca^2+^-binding motifs are present in all ME-7s (DRE and DxNDNxPxF) and ME-13s (DxNDNxPxF), while every ME-18 contains part of a single putative transmembrane domain. Conservation between orthologous alternative exons (a or b) in different species is greater than between paralogous alternative exons (a and b) of the same species, as shown in the cluster, indicating exon duplications before the divergence of insects from Daphnia (Figure 3B). Within each pair, paralogous alternative exons exhibit great sequence diversity from each other. For example, the pair of ME-7s of *Drosophila* N-Cadherin exhibits 50% identity as amino acids, while those of ME-13s and ME-18s display only 47% and 36% identity, respectively. These results indeed suggest that splice-variant isoforms may convey distinct functions. We have previously tested this hypothesis through cell aggregation assays using the *Drosophila* S2 cell line. We revealed that all tested N-Cadherin isoforms are able to mediate heterophilic interactions with each other [41]. The two transmembrane domain isoforms, 7b-13a-18a and 7b-13a-18b, mediate graded homophilic interactions [43], suggesting their potential roles in regulating differential affinity during development, which still awaits *in vivo* testing. Genetic mosaic analyses have demonstrated that by supplying a single isoform of N-Cadherin, morphological defects of N-Cadherin deficient neurons in either visual or olfactory system of adult *Drosophila* brains can be reverted cell-autonomously [34],[38],[39],[41],[44].

**Figure 4:**
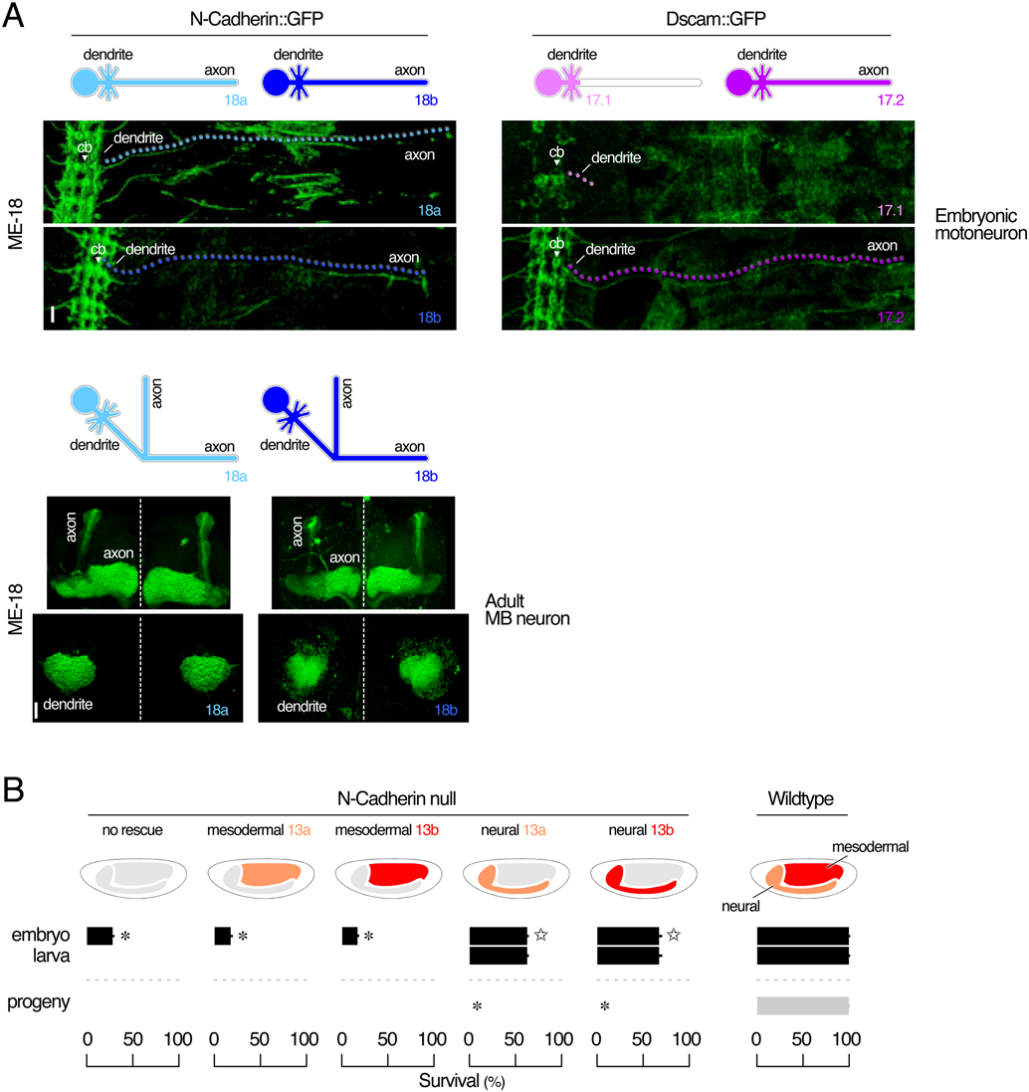
Functional redundancy of splice-variants in *Drosophila*. (A) Subcellular localization of ectopically expressed GFP-tagged N-Cadherin and *Dscam* splice-variant isoforms in the wildtype background. GFP-fused cDNAs of N-Cadherin isoforms derived from *ME-18a* and *ME-18b* are expressed in embryonic motoneuron (upper left panel, *eve'-GAL4^RN2^*), and adult mushroom body interneurons (lower left panel, *GAL4^201Y^*). GFP-fused cDNAs of *Dscam* isoforms derived from *ME-17.1* and *ME-17.2* are expressed in embryonic motoneuron (upper right panel, *eve'-GAL4^RN2^*). Dotted lines in the upper panels trace protein localization in axons. Vertical dotted lines in the lower panel indicate the midline of the brain. cb, cell body. Scale bar in the upper panel 10 µm, in the lower panel 30 µm. (B) Genetic rescue of N-Cadherin null mutant through expression of cDNAs of isoforms derived from either *ME-13a* or *ME-13b* in neural (*elav'-Gal4*) and mesodermal (*Gal4^24B^*) tissues. Asterisk indicates a significant reduction in viability as compared to wildtype control at p<0.01 with two-tailed t-test. Star indicates a significant reversion of the lethality over the null. Note that null mutants with the neural expression of *ME-13a* and *ME-13b* derived isoforms survive through adult stage but fail to produce progeny.

We conducted two additional approaches using *Drosophila* neurons as the *in vivo* model system to evaluate whether splice-variant isoforms exhibit diverse functions. First, we considered the possibility that transmembrane domain splice-variants might be targeted to distinct subcellular compartments of neurons, as is the case with Dscam [19],[20], where they would serve specific functions. Neurons are polarized cells. They contain distinct compartments including a single axon and several dendrites. Each distinct compartment is molecularly, structurally and functionally different from each other. We generated GFP-tagged N-Cadherin transmembrane domain isoforms (7b-13a-18a::eGFP and 7b-13a-18b::eGFP) and then drove their expression in either adult mushroom body interneurons or embryonic motoneurons. In either type of neuron, both isoforms are localized to dendrites and axons, showing no obvious protein targeting bias (Figure 4A). Moreover, the localization of ectopically expressed GFP-tagged isoforms is similar to that of the endogenous protein visualized by immunostaining, although 7b-13a-18a::eGFP localized more specifically to the synapses in the motoneuron axons. Second, we wanted to test the differential ability for N-Cadherin splice variants to rescue the viability of genetically N-Cadherin null animals. The loss of endogenous N-Cadherin leads to embryonic lethality [36]. We reasoned that if individual isoforms possess unique features that cannot be substituted by others, they would exhibit different abilities in rescuing the embryonic lethality in null mutant embryos. This simple test has the potential to reveal critical functional differences among the isoforms even when morphological criteria might fail. We used splice-variants derived from alternative use of ME-13s, i.e., 7b-13a-18a and 7b-13b-18a. When expressed transgenically in neurons of the N-Cadherin null mutants, either isoform is capable of partially rescuing the lethality (Figure 4B), similar to expression in both neurons and mesoderm (data not shown). However, when expressed transgenically in mesoderm of the N-Cadherin null mutants, neither isoform rescues the lethality. Expression levels of transgenic proteins are at least as high as those of endogenous N-Cadherin. This does not induce any visible gain-of-function phenotype (data not shown). In summary, our *in vivo* results showed no differential subcellular localization between transmembrane domain isoforms containing exons 18a or 18b in *Drosophila*. Moreover, there are no functional differences in rescuing embryonic viability between extracellular Cadherin domain isoforms containing exons 13a or 13b. Although we could not rule out the possibility that these tests are unable to distinguish functional differences between splice-variants, *in vivo* data from our lab or other labs [34],[38],[39],[41],[44] do not support the hypothesis that N-Cadherin splice-variants possess diverse functions as proteins.

### Nucleotide sequence conservation of arthropod N-Cadherin

**Figure 5:**
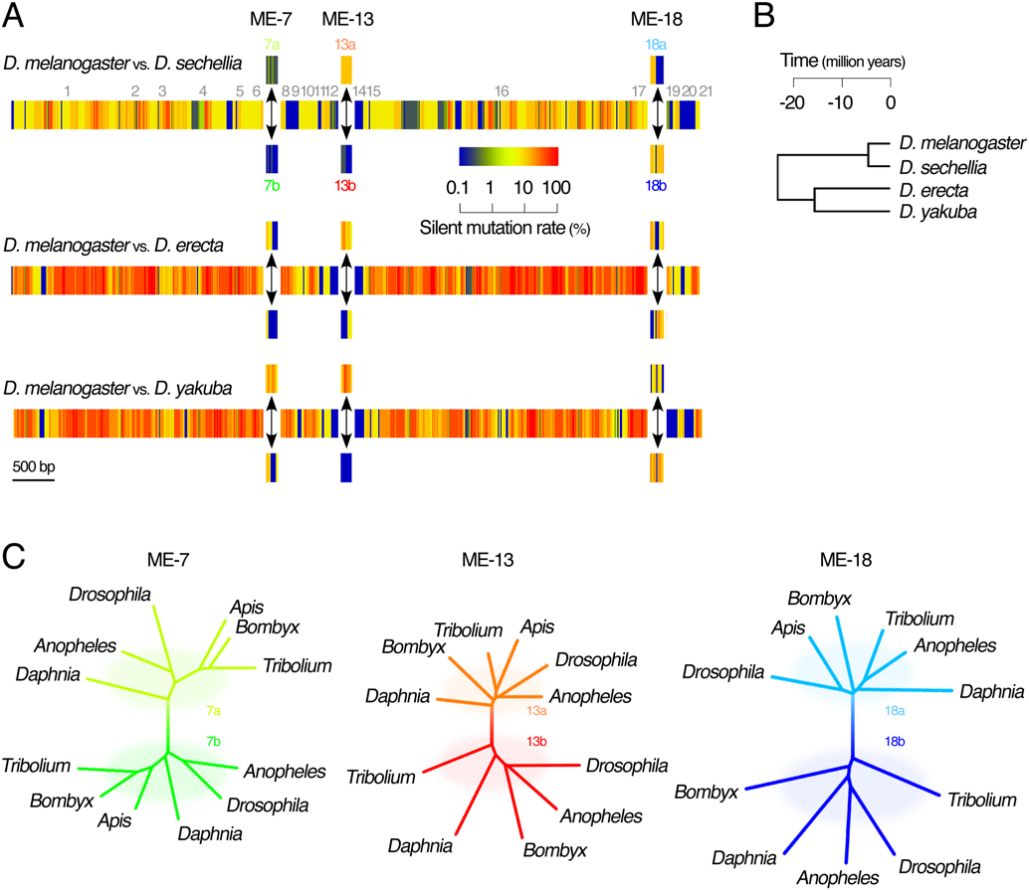
Conservation of nucleotide sequences in MEs. (A) Synonymous mutation rates of mRNAs of *N-Cadherin* isoforms between *Drosophila* species. Exon numbers are shown on the top of the graph. The heat bar graphs are presented in a log scale with low silent mutation rates in blue and high ones in red. All mRNA sequences containing the same alternatively spliced exons were aligned and sliding window analysis of synonymous mutations was performed with Swaap 1.0.2 program basing on Li's method [46], with a window size of 90 nucleotides and a step size of 18 nucleotides. In ME-7s, *ME-7a* and, to a lesser extent, *ME-7b* exhibit unusually low rates. In ME-13s, *ME-13b*, as well as *exon-12* and *exon-14* that surround it, show extremely low rates. In ME-18s, *ME-18a*, *ME-18b* and/or *exon-19* are conserved at the nucleotide level. See Figure S1 for the Ks plots of the same set of *N-Cadherin* mRNAs from the *Drosophila* species on a log scale. (B) Phylogenetic tree of *Drosophila* species. (C) Phylogenetic trees of the *MEs* based on nucleotide sequences (see Figure 3B for comparison).

In addition to serving diverse protein functions, regulated tissue-specific and developmental stage-specific expressions of splice-variants have been shown to be vital to their functions. If the ME-containing transcripts of N-Cadherin were to receive distinct tissue-specific splicing regulation at the nucleotide level, then the MEs themselves as well as adjacent common exons would exhibit a higher degree of evolutionary conservation due to the presence of cis-acting regulatory elements [22], [25]–[27]. In order to distinguish nucleotide conservation because of functional constraints on nucleotide sequences from that on the protein-coding sequences, we first conducted an analysis based on the relative frequencies of nonsynonymous and synonymous (silent) mRNA mutations. We limited our pool of genomes to closely related species within the *Drosophila* genus that are less than 25 million years apart to avoid potential skewing of data by multiple independent silent mutations at a given locus (Figure 5B) [45]. This analysis shows that nonsynonymous mutations are close to or equal to zero among orthologs of *Drosophila* N-Cadherin. In addition, it reveals an extraordinarily low synonymous mutation rate in and near the MEs of N-Cadherin, suggesting the presence of conserved cis-regulating elements. The plummet of synonymous mutation rate is more apparent in ME-7s and ME-13s than in ME-18s. This finding further implies that their splicing might be regulated in a similar fashion in *Drosophila* and *Tribolium* (Figure 5A and Figure S1). Second, we also noted that in some cases the nucleotide sequences of orthologous exons cluster tighter than their amino acid sequences, in particular, ME-13b, and to a lesser extent also ME-18b (compare Figure 5C to Figure 3B). This implies relatively high selective pressures on the nucleotide sequences within these MEs in arthropod genomes. Thus, our *in silico* analyses suggested evolutionary conservation of diversified splicing regulatory regions for the N-Cadherin gene, especially for the ME-13s.

### Conserved expression patterns of arthropod N-Cadherin splice variants

**Figure 6:**
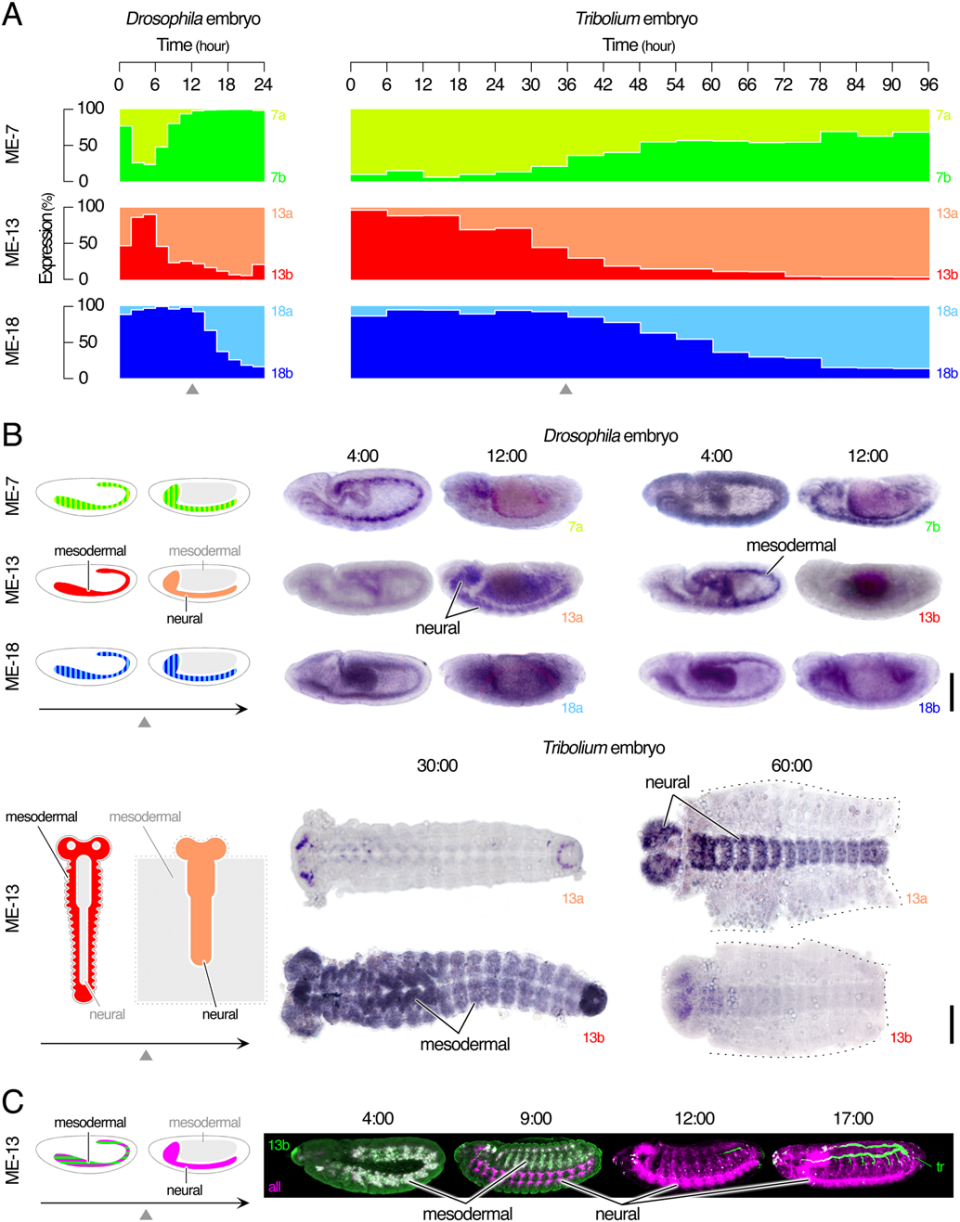
Conserved spatiotemporal expression pattern of MEs. (A) Relative abundance of ME usage in mRNAs during embryogenesis in *Drosophila* and *Tribolium* by quantitative RT-PCR. Note that maternally supplied mRNA is abundant during hours 0–2 in *Drosophila*. Synaptogenesis begins after hour 9 in *Drosophila* and hour 36 in *Tribolium* (*triangles*). (B) In situ hybridization of MEs in *Drosophila* (lateral views) and *Tribolium* (dorsal views) embryos. *ME-13a* and *ME-13b* are used in “'neural” and “mesodermal” splice-variants, respectively. *Tribolium* embryos at 60:00 are fillet-dissected (dotted line). (C) Immunocytochemistry of *ME-13b* derived isoforms (green) in *Drosophila* embryos, counterstained with antibody that labels all N-Cadherin (magenta). The isoform-specific antibody shows non-specific binding of trachea. Scale bars 100 µm.

Highly conserved sequences in and around the MEs further suggest conserved spatiotemporal regulation of alternative splicing of arthropod N-Cadherin splice-variants during evolution, which we examined further by conducting the following *in vivo* tests. First, we used real-time PCR to quantify the dynamic temporal regulation of the MEs during embryogenesis of *Drosophila* and *Tribolium* (Figure 6A). We found that, for each of the three sets of MEs, a switch of predominant ME occurs similarly in the two insects that are separated by 290 million years. The timing of switches between different ME pairs is not precisely parallel (compare, for example, ME-7s and ME-18s), suggesting separate regulation. Second, we examined *in situ* spatiotemporal expression patterns of the three pairs of MEs in *N-Cadherin* of *Drosophila* and *Tribolium*. Our results revealed expression of ME-7a, ME-7b, ME-18a and ME-18b in both the embryonic CNS and the early mesoderm (Figure 6B). However, ME-13a is detected only in the CNS, while ME-13b is only expressed in the early mesoderm. Furthermore, the non-neuronal expression of ME-13b drops sharply before synapses begin to form in the embryos (Figure 6A and 6B, triangles). Third, we raised an isoform-specific antibody against *Drosophila* ME-13b to determine whether the same spatiotemporal regulations of ME-13s are reflected at the protein level (Figure 6C). The antibody detects isoforms containing ME-13b only in the early mesoderm, further confirming distinct expression patterns for the isoforms containing ME-13a and ME-13b. The labeling disappears soon after the mRNA becomes undetectable, indicating rapid degradation of the protein in the mesoderm. As a result, while the nervous system maintains ME-13a-containing “neural” N-Cadherin isoforms, there is little N-Cadherin protein in the muscles of late-stage embryos or early-stage larvae. Taken together, our *in vivo* data showed conserved spatiotemporal expression patterns between orthologous MEs, supporting the model of N-Cadherin isoforms receiving distinct and evolutionarily conserved expression regulation.

**Figure 7:**
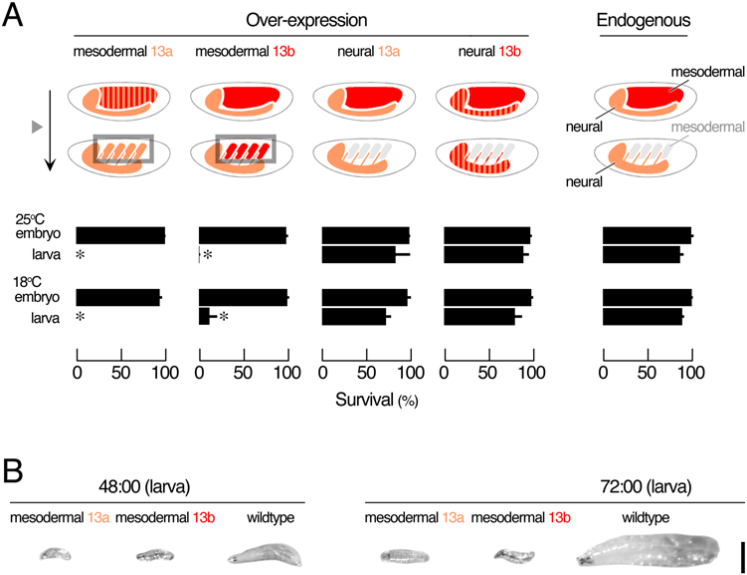
Detrimental effects of overriding the endogenous spatiotemporal control. (A) Over-expression of *ME-13a*- and *ME-13b*-containing isoforms in neural (*elav'-Gal4*) or mesodermal (*Gal4^24B^*) tissues at 25°C and 18°C. Ectopic expression of either isoform in the nervous system or mesoderm (*box*) has no significant effect during embryogenesis (one way ANOVA, p = 0.39 at 25°C and p = 0.03 at 18°C). However, expression of either isoform in mesoderm beyond the onset of synaptogenesis (*triangle*) caused lethality in larvae (two-tailed t-test, p<0.001 for either isoform at both temperature). In contrast, expression of either isoform in the nervous system had no significant effects on the survival either in embryonic or larval stage (two-tailed t-test, p>0.001 for either isoform at both temperature). Asterisk indicates a significant reduction in viability as compared to wildtype control at p<0.001 with two-tailed t-test. (B) Over-expression of either isoform in mesoderm causes muscle paralysis in larvae. Scale bar 500 µm.

We reasoned that if maintaining the spatiotemporal regulation of N-Cadherin splicing were essential, then deliberately deviating from the endogenous pattern of N-Cadherin expression would cause detrimental effects to the survival of an organism. Using *Drosophila*, we ectopically expressed the cDNA of a N-Cadherin isoform containing ME-13b (7b-13b-18a) in muscles of wildtype *Drosophila* (Figure 7A) at standard temperature (25°C). Devoid of introns, the transgene is free from endogenous splicing regulation and can be expressed in the mesoderm beyond the point at which endogenous N-Cadherin expression ceases, thus allowing the prolonged expression of these splice variants. This results in no change in the survival rate during the embryogenesis but a robust (95.5%) lethality during larval stages. The larvae exhibit reduced locomotion and remain small in size prior to their death (Figure 7B) but display no apparent abnormality in the muscle morphology (data not shown). Interestingly, despite its considerable amino acid divergence, the cDNA of another N-Cadherin isoform containing ME-13a (7b-13a-18a) proves to be an equally potent agent of lethality (100% lethality). The lethal stage caused by temporal mis-expression of splice variants is distinct from that caused by genetic deletion of the *N-Cadherin* gene, which occurs during late embryonic stages and with neuronal pathfinding defects [36]. Since deletion of the *N-Cadherin* also causes neural pathfinding defects, we ectopically express N-Cadherin cDNA containing either ME-13a (7b-13a-18a) or -13b (7b-13b-18a) continuously in the CNS, where endogenous ME-13a-containing isoforms are expressed. We found this induces no abnormality throughout both the embryonic and larval stages (Figure 7A). To distinguish whether the elevated levels or the extended temporal expression of DN-Cadherin in muscles is the main cause of the lethality phenotype in larvae, we reduced the level of exogenous N-Cadherin expression by raising the animals at a lower temperature (18°C). Total amount of N-Cadherin (both endogenous and exogenous) 7–9 hours after egg-laying was quantified by western blot (data no shown). At 25°C, the exogenous expression levels of 13a- and 13b-containing isoforms are 14 and 9 folds of the endogenous N-Cadherin level, respectively. At 18°C, it dropped to approximately 3 folds of the endogenous N-Cadherin level. Despite the 3–5 folds drop of exogenous expression of 13a- and 13b-containing isoforms at 18°C, the lethality of larvae remains the same as that at 25°C (Figure 7A). Thus, down-regulation of N-Cadherin expression in muscle cells beyond 12 hours after egg-laying is essential to the normal development of *Drosophila* embryos.

In this report, we discover that the expression of *N-Cadherin* splice variants is spatiotemporally regulated during development. At embryonic stage *in vivo*, despite that *N-Cadherin* contains the “neural” and “mesodermal” splice-variant isoforms, the protein products of these splice variants are functionally interchangeable. However, prolonged expression of *N-Cadherin* splice isoforms after embryonic stage in the muscle causes lethality whereas the prolonged expression in neurons causes no adverse effect. These genetic manipulations in *Drosophila* offer *in vivo* evidence to support the significance of the spatiotemporal regulation of expression of N-Cadherin splice-variant isoforms. In addition, the three sets of MEs of arthropod *N-Cadherin* and the spatiotemporal regulation of the spliced variants are conserved over 400 million years. Therefore, we hypothesize that alternative splicing of *N-Cadherin* is critical for arthropod embryonic development and it provides the complexity required for developmental regulation.

## Discussion

### Alternative splicing of N-Cadherin is conserved in arthropod genomes

Metazoan development requires the collaboration of many morphologically and functionally distinct cell types, resulting from differential gene expression patterns. A separate set of mRNA molecules in each individual cell is generated through regulation at several different levels, including transcription and splicing. Combinatorial expression of transcription factors has been shown to dictate cell fates, while various RNA-binding proteins could generate multiple splice-variant isoforms from a single species of pre-mRNA molecules [10].

*Drosophila* N-Cadherin belongs to the classic Cadherin family. Through inspection of several recently released arthropod genomes, we showed that the genomic structure of three pairs of mutually-exclusive alternatively-spliced exons, or MEs, of *N-Cadherin* has been conserved in the arthropod lineage for more than 400 million years. The only exception is the recent loss of ME-13b and ME-18b after the divergence of *Apis mellifera* from other insects. MEs of the arthropod *N-Cadherin* gene encode part of protein domains composed of diverse amino acid sequences while maintaining the same overall structure.

### Alternatively spliced N-Cadherin proteins are functionally redundant

Alternative splicing has been considered to be an important means to generate diverse protein products from a single gene, thus expanding the proteome from limited amount of genetic material. Using RT-PCR, we confirmed the endogenous expression of all MEs in *Drosophila melanogaster* and *Tribolium castaneum* embryos. The small number of splice-variants in arthropod *N-Cadherin* gene has offered an opportunity to experimentally evaluate whether resulting isoforms adopt distinct protein functions to expand protein diversity and/or receive separate spatiotemporal controls leading to increased complexity in expression patterns.

*Drosophila* N-Cadherin isoforms have been shown to exhibit differential homophilic binding affinity when expressed in *Drosophila* S2 cells [43]. However, a number of studies have demonstrated that re-supplying a single N-Cadherin isoform could cell-autonomously rescue morphological defects of N-Cadherin-deficient neurons in adult *Drosophila* brains [34],[38],[39],[41],[44]. Consistent with this, our own results showed that transmembrane-domain splice-variants (ME-18s) have no targeting preference to different subcellular compartments of neurons. We also showed that one pair of extracellular Cadherin domain isoforms (ME-13s), despite their distinct tissue-specific expression patterns (ME-13a being neuronal and ME-13b being mesodermal), show no differences in their ability to cell-autonomously partially rescue embryonic lethality caused by genetic loss of *N-Cadherin* gene (Figure 4B). Thus, results from *in vivo* tests in *Drosophila* do not support the idea that N-Cadherin splice-variants provide significant functional diversity as proteins during development.

### Alternatively spliced N-Cadherin variants are expressed in a complex and essential pattern

Our *in silico* analysis of mRNA sequences of *N-Cadherin* splice-variants revealed extremely low synonymous mutation rates at MEs and/or flanking constitutive exons (Figure 5A) and a tight clustering of nucleotide sequences (Figure 5C). These results imply a heightened conservative selective pressure on the local nucleotide sequences. Another independent line of evidence comes from our *in vivo* observation of highly conserved endogenous ME expression patterns. *Drosophila* and *Tribolium* diverged from each other 290 million years ago. They undergo different modes of embryogenesis (i.e., short germ-band vs. long germ-band, respectively), and for different durations (i.e., 24 hours vs. 96 hours, respectively). Nevertheless, the overall spatiotemporal patterns in usage of individual MEs in these two distant insect species are extremely similar (Figure 6A and 6B).

Using *Drosophila*, we designed *in vivo* experiments to examine the significance of the evolutionarily conserved spatiotemporal regulations of the alternative isoforms. We found that when N-Cadherin protein expression in muscles is abnormally prolonged only by several hours, the fitness of the organism is in serious jeopardy, regardless of the isoform expressed (Figure 7). Deliberate deviation from the normal spatiotemporal restriction on N-Cadherin expression leads to a robust lethality. This occurs when the molecule is delivered to the right tissue (i.e., the muscles), but at the wrong time (i.e., after synaptogenesis). On the other hand, as long as it is expressed within the precise spatiotemporal constraint, over 50% amino acid divergence within a functional domain of N-Cadherin proteins can be tolerated even at levels comparable to the endogenous expression. These isoforms may be functionally distinct, however, their significant aspects are not yet obvious and are difficult to test. Our results support the idea that the regulated expression of arthropod N-Cadherin MEs generates a complex but essential pattern of spatiotemporal expression of alternative isoforms. In conclusion, our *in silico* and *in vivo* analyses of the arthropod N-Cadherin presents an example in which well conserved alternative splicing increases the spatiotemporal expression complexity essential for metazoan development.

## Materials and Methods

### Analysis of genomic sequences

The intron sequences of the *Drosophila N-Cadherin* gene (*CadN*, Gene Bank AB002397) were subjected to BLASTX (NCBI) search of the *Drosophila melanogaster* protein database to identify potential alternatively spliced exons. The possible open reading frames/exons within these introns were further analyzed for the presence of proper splicing donor and acceptor sites, and for the maintenance of correct phase and orientation. Alternatively spliced exons occur as three modules in the structure-coding sequence. Previously, Iwai et al. had isolated two cDNA isoforms that contain, respectively, the 7a-13a-18a (CG7100-PE) and 7b-13a-18a (CG7100-PD) combinations, and have further characterized the latter. A 12-nucleotide long micro exon-7a' is also predicted in all insect genomes examined in this study (data not shown).

### N-Cadherin orthologs

The N-Cadherin orthologs analyzed are annotated based on homology with CG7100 (*Drosophila melanogaster*) from the following genomic sequences: CH480822 (*Drosophila sechellia*), CM000158 (*Drosophila yakuba*), CH954179 (*Drosophila erecta*), CH902620 (*Drosophila ananassae*), gnl| dpse| 4_group3 (*Drosophila pseudoobscura*), CH479187 (*Drosophila persimilis*), CH963913 (*Drosophila willistoni*), CH933807 (*Drosophila mojavensis*), CH940649 (*Drosophila virilis*), CH916368, CH916433, CH916679 (*Drosophila grimshawi*), AAAB01008980 (*Anopheles gambiae*), CH477197 (*Aedes aegypti*), CH381939, CH384411, CH388564, CH386188, CH386289 (*Bombyx mori*), NW_001253165 (*Apis mellifera*), NW_001092836 (*Tribolium castaneum*) and scaffold_100 (*Daphnia pulex*).

### Silent mutation rate

*N-Cadherin* mRNA sequences from *Drosophila sechellia*, *Drosophila yakuba*, *Drosophila erecta* and *Drosophila ananassae* were selected to conduct silent (synonymous) mutation analysis because of their relatively short evolutionary distance from *Drosophila melanogaster*. *Drosophila simulans* was rejected from the analysis because of a sequencing gap within the coding region of N-Cadherin while *Drosophila ananassae* was rejected due to saturation of the synonymous sites. All transcripts containing the same alternatively spliced exons were aligned and sliding window analysis of the synonymous mutation rate [40] was done with Swaap 1.0.2 program [46], with a window size of 90 nucleotides and a step size of 18 nucleotides. Nonsynonymous mutations were extremely rare among the *Drosophila* species. Therefore, the nonsynonymous mutation rate (Ka) is equal to zero in most of the windows. Because of that, instead of Ka/Ks, we plotted the Ks on a logarithmic scale.

### Phylogenetic trees

The phylogenetic trees of MEs in the arthropod genomes were constructed from their amino acid sequences (Figure 3B) and nucleotide sequences (Figure 5C) using Clustal W and DRAWTREE (PHYLIP unrooted phylogenetic tree) with Biology workbench (http://seqtool.sdsc.edu).

### Fly stock

*Drosophila melanogaster* cultures were kept in standard media at 25°C. Embryogenesis takes 24 hours at room temperature. For RT-PCR reactions, the *w^1118^* strain flies were placed in a cage and allowed to lay eggs on grape juice plates with supplemental yeast paste for certain egg-laying periods. The plates were placed at room temperature (25°C) before reaching specified hours after egg-laying. Embryos were rinsed with water and then frozen with dry ice. For larvae collection, wandering third instar larvae were collected, washed with PBS, and frozen with dry ice. The mutant analysis on N-Cadherin loss-of-function was based on *Ncad ^405^* (source: L. Zipursky) and *Ncad^M19^* (source: T. Uemura). The *elav'-GAL4* and *GAL4^24B^* drivers (source: C. Goodman) were used to drive *UAS-Ncad^7b-13a-18a^* and *UAS-Ncad^7b-13b-18b^* (source: C-H. Lee and A. Chiba) expression in neurons and in muscles, respectively. The *eve'-GAL4^RN2^* (source: M. Fujioka and J. Jaynes) and *GAL4^201Y^* (source: T. Lee) drivers were used for cell-specific fluorescent labeling with *UAS-dscam^17.1^::GFP* , *UAS-dscam^17.2^::GFP* (source: J. Wang), *UAS-Ncad^7b-13a-18a^::GFP* or *UAS-Ncad^7b-13a-18b^::GFP* (source: C-H. Lee and A. Chiba).

### Beetle stock

*Tribolium castaneum* cultures were kept in standard media at 29°C. Embryogenesis takes 96 hours. For quantitative real-time PCR, after an egg-laying period of 6 hours, the embryos were collected and then kept at 29°C before they reached specified hours after egg-laying. The embryos were dechorionated with 50% bleach that also removes flour particles covering them, frozen with dry ice and kept at −20°C until later processing.

### RNA isolation and reverse transcription (PCR and nested PCR)

Total RNA was isolated using the RNeasy column (Qiagen). Reverse transcription reactions were primed using random hexamers. All PCRs were performed using specific primers and standard protocol or the Advantage cDNA PCR kit (BD Biosciences). The sequences (from 5′ to 3′) of the primers are: (A) cgatctggaatactttgagattcaggctgagtccg, (B) ccgcttgaccgtgaccgttatcgattgagggttag, (C) cgcgagacatacaagttagaggccatggcccaaga, (D) tgcggttaaaaatatgacgggtgccatttatgtag, (E) ggtatgaactccgtttggccgcctccgacaatttg, (F) gacgccggtacgaactgaaattggttgcctcggac, (G) cgatccgttcgagtgcgtagacctgtggaatgtct, (H) ccgaacttgccttaaccgatgttacaaccgtaccc, (I) cgatacccgaactggctttgatcgagacgactttg, (J) cgtaacctgcaagacgcgtttgggcaaattacgat, (K) taacctgcataatggtaaagggcgtgttggtcatt, (L) caagacgagtatcagtatgatcaaaaggcagacca, (M) aagacgagtagtatcagagtgcccaggcagagcgc. The sequences (from 5′ to 3′) of the primers for *Tribolium* are: (N) accaatattctgcgcgtttc, (O) cgagatcacaagcggtaaca, (P) aacttggaacgcaattgtcc, (Q) gtacgtgttcacgtcccaca, (R) gcgatccaccacgtctatct, (S) ctcttcggcagattcctgtc, (T) tgcatcagaagcatgggata, (U) catgctgagcttgagggttt, (V) gtgtaaccggaaggacagga. Nested PCR used the purified PCR products from first round of amplification as the templates to exclude the cDNAs not amplified with desired primer pairs.

### Quantitative real-time PCR

Total RNA was isolated using TRIZOL (Invitrogen) and reverse-transcribed with the Thermoscript III RT system (Invitrogen). Random hexamers were used to synthesize cDNA from whole animal RNA samples. We used 100 ng of reversely transcribed product for each TaqMan™ real-time PCR analysis. Primers and TaqMan probes were designed using PrimerExpress 1.0 software (PerkinElmer Life Sciences). Each assay was designed to detect one specific alternative exon and contained one general primer and one exon-specific primer to amplify 100–150 bp of the *N-Cadherin* transcript. To increase specificity, the designed TaqMan probes encompassed the junction sequence between the alternative and common exons (see below for primer sequences). TaqMan probes, primers, and universal TaqMan master mix were obtained from Applied Biosystems and used according to the manufacturer's instructions. An ABI Prism 7000 Sequence Detection System was used for real-time PCR analyses. Controlled amounts (1 to 1000 fg) of *N-Cadherin* isoform cDNAs were used as templates to derive standard curve and PCR efficiency, and to test cross-reactivity for each assay. To construct the standard curve for the internal control, 18S rRNA, we used 1 to 1000 fg of the cDNAs. The following thermo-cycling program was used for PCR amplification: (i) one cycle at 50°C for 2 minutes and 95°C for 10 minutes, (ii) 50 cycles at 95°C for 15 seconds and 60°C for 1 minutes, and (iii) holds at 4°C. Experiments were performed in triplicate. The data were analyzed using ABI Prism 7000 software. Standard errors of the ratio were calculated using the delta-method with the following formulation: 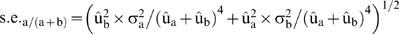 where 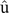 and σ are the sample mean and standard error, respectively. The (i) forward (5′–3′) primers, (ii) reverse (5′–3′) primers and (iii) TaqMan probes used for *Drosophila* real-time PCR are as follows. ME-7a: (i) tgtcactgtgggtacggttgtaa, (ii) tcgataaaaaactgtgggattgc, atcggttaaggcaagttcgggtatcgaa. ME-7b: (i) cccgttggcggcaaa, (ii) actgtgggattgccttcgat, (iii) tcgtctcgatcaaagccagttcggg. ME-13a: (i) ccagattaccgaagaggacgat, (ii) cgatccttgtcgccatctgt, (iii) tgcccaaacgcgtcttgcaggtta. ME-13b: (i) tcgacgagggaatgaccaa, (ii) cgatccttgtcgccatctgt, (iii) acgccctttaccattatgcaggttacgg. ME-18a: (i) cgtagacctgtggaatgtctacga, (ii) ccttggaatcgggtgacataa, (iii) tgcacttgcggcgaaggacg. ME-18b: (i) cgtagacctgtggaatgtctacga, (ii) tgctgcccgagcttttgt, (iii) tgcacctgtccggcgggc. The (i) forward (5′-3′) primers, (ii) reverse (5′-3′) primers and (iii) TaqMan probes used for *Tribolium* real-time PCR are as follows. ME-7a: (i) cgagaaaatgtcacagtgggtact, (ii) gaaaaccgtcggattgttctct, (iii) ttcggtgaaggccagtt. ME-7b: (i) ttacggaccgatcttcattcg, (ii) aaccgtcggattgttctctatacc, (iii) tgtccgtcaaggccagt. ME-13a: (i) cgaagaggacgacaggaatctg, (ii) cccgtcaggaagtagacaatgtt, (iii) ctcagggtgacggcaa. ME-13b: (i) aagagtatccggctccctatcc, (ii) cccgtcaggaagtagacaatgtt, (iii) tgatgcaggtgacggca. ME-18a: (i) gccgccattctagtctgctt, (ii) gcgtctgttgtacaccacgaa, (iii) ctcatgattttgatcttagtgttg. ME-18b: (i) cgtccggagcgatcatg, (ii) gcctcacggcgtctgttg, (iii) acttctagtcttagtgttggtatt. 18S rRNA: (i) tcgctagctggcatcgttt, (ii) gaggttcgaaggcgatcaga, (iii) tggttagaactagggcggt. The experiments shown in Figure 6A were repeated twice.

### RNA *in situ* hybridization

The *Drosophila* protocol was based on the 96-well hybridization protocol of BDGP (http://www.fruitfly.org/about/methods/RNAinsitu.html) and the *Tribolium* protocol was modified by Yoshinori Tomoyasu from what has been described before [47]. In brief, individual exons were amplified by PCR and then cloned into pCRII-TOPO vectors or pCRIV-TOPO vectors (Invitrogen). RNA probes of specific exons were made following the digoxygenin RNA labelling Kit protocol (Roche Molecular Biochemicals). *Drosophila* RNA probes for exon-7a and exon-7b are about 300 nts long, which include the specific exons and the flanking exonic sequences. *Drosophila* RNA probes for exon-8, exon-13a, exon-13b, exon-18a and exon-18b contains only the designated exons, respectively. *Tribolium* RNA probes are all about 500 nts long, including the designated exons and the flanking exonic sequences. Embryos were collected for 24 hours at 25°C for *Drosophila* and 96 hours at 29°C for *Tribolium*, dechorionated, and then fixed with freshly made 4% paraformaldehyde in phosphate-buffered saline. After incubating with hybridization buffer, RNA probes were added to the embryos and allowed to hybridize overnight at 55°C. The signal was detected using anti-digoxygenin alkaline-phosphatase (Roche Molecular Biochemicals) and then NBT/BCIP substrate.

### Immunocytochemistry

GST protein fused with seven repeats of oligopeptide (LDEGMTNTPFT) was designed as the antigen to generate the antibody specific to *Drosophila* N-Cadherin derived from ME-13b. The strategy of DNA construction was performed as previously described [48] with minor modification. Briefly, we designed two oligonucleotides. Oligo A (5′-cta gac gaa ggt atg act aac acg ccg ttc acg) encodes the target antigen, and oligo B (5′-gtc ata cct tcg tct agc gtg aac ggc gtg tta) is partly complementary to oligo A. The template-repeated polymerase chain reaction (TR-PCR) method was applied to construct the DNA fragment encoding multiple copies of 13b antigen. To incorporate restriction sites for sub-cloning at both ends of the TR-PCR products (*Bam*HI at the 5′-end and *Eco*RI at the 3′-end) as well as a stop codon at the 3′-end of the coding region, a second round of PCR (adapter PCR) with two adapter primers, primer A (5′-gga tcc cta gac gaa ggt atg ac) and primer B (5′-g aat tca aag ctt cgt gaa cgg cgt gtt) was performed. The DNA fragment encoding the seven repeats of antigen was subcloned into plasmid pGST-KG. The resulting plasmid was introduced into XL-10 Gold. The fusion protein was purified by glutathione-Sepharose 4B affinity chromatography. Rabbits were then immunized with the purified fusion protein and sera were collected.

### GFP-fused transgenes

Two splice-variant isoforms Ncad ^7b-13a-18a^ and Ncad^7b-13a-18b^, which differ in ME-18s corresponding to the transmembrane domain of N-Cadherin, were fused to eGFP (Clontech) at their carboxyl termini. In Figure 4A, each of their expression is driven in either the mushroom body neurons in adult brain (*GAL4^201Y^*) or the aCC and RP2 motoneurons in embryonic ventral nerve cord (*eve'-GAL4^RN2^*). For comparison, two splice-variant isoforms of Dscam with alternative exons encoding the transmembrane domain (*UAS-dscam^17.1(−19+23)^::GFP* and *UAS-dscam^17.2(−19+23)^::GFP)* were expressed in the mushroom body neurons (*GAL4^201Y^*) and embryonic motoneurons (*eve'-GAL4^RN2^*) through parallel crosses.

### Genetic rescue

*Drosophila* embryos were dechorionated and those of desired genotypes were hand-picked. They were later placed on the grape juice plates and kept in the moist chamber at 25°C. The number of hatched larvae was checked after more than 24 hours. The viability of different genotypes was normalized to that of wildtype. Sample sizes (n) in Figure 4B are: 450 individuals (*w^1118^*, *+/+*), 349 (*N-Cadherin null* with *no rescue*, *Ncad^M19^/Ncad^405^*), 340 (rescue with *mesodermal ME-13a*, *UAS-Ncad^ 7b-13a-18a^/+*;*Ncad^M19^/Ncad^405^*;*GAL4^24B^/+*), 174 (rescue with *mesodermal ME-13b*, *Ncad^M19^/Ncad^405^*; *GAL4^24B^/UAS-Ncad^7b-13b-18a^*), 333 (rescue with *neural ME-13a*, *UAS-Ncad^7b-13a-18a^/+*;*Ncad^M19^/Ncad^405^*;*elav'-GAL4/+*) and 210 (rescue with *neural ME-13b*, *Ncad^M19^/Ncad^405^*;*elav'-GAL4/UAS-Ncad^7b-13b-18a^*).

### Overexpression

*Drosophila* embryos were collected at room temperature for two hours on the grape juice plates. Fifty embryos were hand-picked for each replicate, placed on the grape juice plates, and then kept at either 25°C or 18°C. Four replicates are maintained for each genotype at a particular temperature. The developmental time course at 18°C was twice as long as that at 25°C. The number of hatched larvae was quantified at least 24 hrs (25°C) and 48 hrs (18°C) after collection. The number of surviving pupae was checked 5 days (25°C) and 10 days (18°C) after collection. The viability of different genotypes was normalized to that of wildtype. Sample sizes (n) at 25°C are: 187 individuals (*w^1118^*, *+/+*), 190 (over-expression of *mesodermal ME-13a*, *UAS-Ncad^7b-13a-18a^/+*;*GAL4^24B^/+*), 155 (over-expression of *mesodermal ME-13b*, *GAL4^24B^/UAS-Ncad^7b-13b-18a^*), 169 (over-expression of *neural ME-13a*, *UAS-Ncad^7b-13a-18a^/+*;*elav'-GAL4/+*) and 154 (over-expression of *neural ME-13b*, *elav'-GAL4/UAS-Ncad^7b-13b-18a^*). Sample sizes (n) at 18°C are: 187 individuals (*w^1118^*, *+/+*), 186 (over-expression of *mesodermal ME-13a*, *UAS-Ncad^7b-13a-18a^/+*;*GAL4^24B^/+*), 131 (over-expression of *mesodermal ME-13b* , *GAL4^24B^/UAS-Ncad^7b-13b-18a^*), 171 (over-expression of *neural ME-13a*, *UAS-Ncad^7b-13a-18a^/+*;*elav'-GAL4/+*) and 151 (over-expression of *neural ME-13b*, *elav'-GAL4/UAS-Ncad^7b-13b-18a^*). Parental control lines (*elav'-Gal4*, *GAL4^24B^*, *UAS-Ncad^7b-13a-18a^* and *UAS-Ncad^7b-13b-18b^*) were included in parallel during the analysis and yielded similar results to wildtype (data not shown).

### Imaging

Fluorescent images were taken with Zeiss LSM 510 confocal microscope. The three-dimensional projections of Z-stack images were then constructed with Volocity (Improvision).

## Supporting Information

Figure S1 Low synonymous mutation rates at MEs. Plots of synonymous mutation rates of *N-Cadherin* isoforms between *Drosophila melanogaster* and other *Drosophila* species. The Y-axis is the silent mutation rate [40] plotted on the logarithmic scale, while the X-axis is the full length mRNA of *7b- 13a-18a* (upper panel) or *7a-13b-18b* (lower panel). Short horizontal bars indicate the locations of MEs.
(1.12 MB TIF)
