# Supplementary figures and images for: Conserved Alternative Splicing and Expression Patterns of Arthropod N-Cadherin
Source: PLoS Genet. 2009 Apr 3;5(4):e1000441. doi: 10.1371/journal.pgen.1000441 (PMC2655722; doi:10.1371/journal.pgen.1000441)

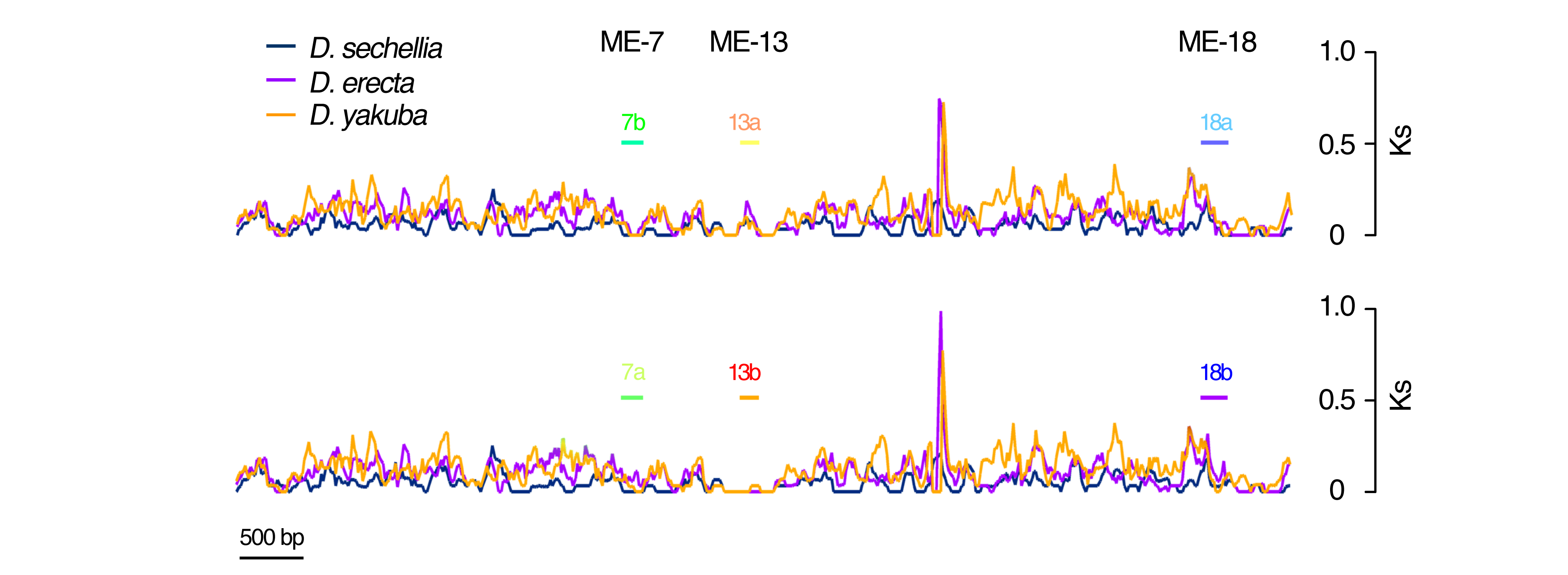

Supplement: Figure S1 — Low synonymous mutation rates at MEs. Plots of synonymous mutation rates of N-Cadherin isoforms between Drosophila melanogaster and other Drosophila species. The Y-axis is the silent mutation rate [40] plotted on the logarithmic scale, while the X-axis is the full length mRNA of 7b- 13a-18a (upper panel) or 7a-13b-18b (lower panel). Short horizontal bars indicate the locations of MEs. (1.12 MB TIF) [file pgen.1000441.s001.tif]
